# Analyzing multi-level governance dynamics from a discourse network perspective: the debate over air pollution regulation in Germany

**DOI:** 10.1186/s12302-022-00640-0

**Published:** 2022-07-14

**Authors:** Melanie Nagel, Carlos Bravo-Laguna

**Affiliations:** 1 Institute of Political Science, University of Tuebingens, Tuebingen, Germany; 2 Department of Political and Social Sciences, Universitat Pompeu Fabra/Institut Barcelona d’Estudis Internacionals, Barcelona, Spain

**Keywords:** Discourse networks, Air pollution, Multi-level governance, Discourse coalitions

## Abstract

**Background:**

Designed to set limits to air pollutant emissions, EU Directive 2008/50/EG has pushed city administrations to strengthen their commitment to environmental protection with mixed results. However, the effectiveness of these policies remains suboptimal. Within this context, this paper provides original empirical evidence on the nature and evolution of the debate over air pollution in the EU that helps readers understand how the interplay among discourse coalitions across levels of governance relates to the implementation of air pollution directives in Europe. To provide this evidence, we apply the discourse-coalition framework to a multi-level governance context by comparing debates over air pollution and examining their evolution in different contexts through the use of discourse network analysis.

**Results:**

Our results show that the debate in the EU institutions is largely dominated by pro-environmental coalitions, whereas discussions in Stuttgart and Berlin show different degrees of permeability to such arguments. Hence, the relative prominence of certain actors and levels of governance in the local discourse helps explain the extent to which debates at the local level align with the arguments put forward by the EU institutions. For their part, governmental organizations have a tendency to act as bridges between discourse coalitions.

**Conclusions:**

This novel empirical evidence provides clues that help make sense of the varying levels of implementation of EU air pollution directives at the local level. In this regard, the ability of supranational and national bodies to align their discourse with those of local organizations might facilitate a common understanding and the successful implementation of EU policies. Policymakers aiming to improve air quality at the local level may want to consider engaging actively with this debate by reinforcing the arguments of pro-environmental coalitions. They may also want to make a special effort to ensure that the messages conveyed to supranational and national bodies are also effectively conveyed to lower levels of governance.

**Supplementary Information:**

The online version contains supplementary material available at 10.1186/s12302-022-00640-0.

## Introduction

Beyond its effects on climate change and biodiversity, air pollution poses important risks to human health. Among these, there is widespread evidence of a positive correlation between high air pollution levels and severe cases of COVID-19 [14]. In addition, research suggests that air pollution aggravates cases of cancer, neurological conditions, and immunological deficiencies [35]. Accordingly, Directive 2008/50/EG set limits on air-pollutant emissions at the EU level. In 2018, the European Commission sued Germany and six other member states for non-compliance with its provisions. These events have pushed local administrations to take more effective measures to reduce emissions, since stricter limits at the EU level create pressures for action at the local level. Still, some cities would rather take legal action against the EU, or risk being fined, than adopt the necessary measures to reduce particulate matter pollution [53]. Previous research [24, 32] has highlighted the ineffectiveness of air quality policies in Europe, partly due to deficiencies during the implementation phase.

With this context in mind, this paper provides original empirical evidence on the nature and evolution of the debate over air pollution in the EU that helps make sense of the varying levels of implementation at the local level of EU air-pollution directives. Bearing in mind that this policy area leaves considerable leverage for subnational authorities to intervene in the policymaking process [5], this paper fulfills its purposes by applying the discourse-coalition framework to a multi-level governance context. Hence, it contributes to the existing literature by analyzing the debate around air pollution in Europe from a promising and widely overlooked perspective. More specifically, our approach contributes to understanding how the interplay among discourse coalitions across levels of governance relates to the implementation of air-pollution directives in Europe. The emergence of competing framings in an otherwise socially constructed debate [19] makes the issue of air pollution particularly interesting for our purpose.

This study examines the nature and evolution of the debate around air pollution in the EU through a network lens. In particular, it uses the method of discourse network analysis (DNA) to compare debates in different contexts, to trace storylines, and to identify the roles played by the actors that participate in the abovementioned discussions. The use of this methodological approach helps us to identify the relational dynamics that underlie multi-level governance systems. While several studies have examined policy implementation of air-pollution regulation in Europe [4, 24], few have done so from a discursive perspective. Consequently, we do not know which arguments or narratives are put forward at the different levels involved. Thus, we will shed light on the evolution of discursive tensions across levels of governance.

Our analysis pays special attention to local and EU levels due to their roles in environmental policy. While the local level is key for the effective implementation of air-pollution directives, the EU supranational institutions are in charge of initiating and adopting such directives, as well as of monitoring their implementation [24]. Germany, a central country for air-pollution control in Europe, will be the focus of our study. We have selected influential (Berlin) and extreme (Stuttgart) city cases to analyze the air-pollution debate [49]. In particular, we compare how German newspapers have portrayed the discussions on air pollution in each of these cities in their framing of the debate in the EU institutions.

We have organized this paper as follows. The next two sections introduce our theoretical framework, which examines the multi-level governance of air pollution in Germany from a discursive perspective. “Methodology” section explains the methodology of our study. “Analysis and results” section presents the results of our analysis, whereas “Discussion and conclusions” section discusses our central findings and draws our main conclusions.

## The multi-level governance of air pollution in Germany from a discursive perspective

We will examine the nature and evolution of the discourse around air pollution in Germany through the lens of multi-level governance. While the idea of multi-level governance has been presented in different ways in the literature [57], we refer to it as a concept that accounts for the loss of a national government’s monopoly over policymaking and the subsequent transfer of authority across different levels of governance [30]. More specifically, power flows from the national government in two directions: upward to supranational institutions, and downward to regional and local bodies. The presence of the word “governance” indicates the prominence that this theoretical perspective grants to non-state actors in the policy process [57]. Multi-level governance argues that policymaking takes place in different contested arenas [40], whereby the national, subnational, and supranational levels are immersed in Gramscian struggles for power, and different actors push for advancing their own normative agendas [16]. Originally focusing on the case of the EU [40], multi-level governance studies have expanded their scope to other polities across the world [57].

Political regulations are outcomes of negotiation processes that manifest themselves in dynamic political discourses at different levels. Bearing this in mind, the literature has argued that multi-level governance has considerable policymaking advantages. For example, the *decentralization* of competencies gives municipal administrations room for maneuver to address policy issues whose roots are found at the local level, thus increasing the effectiveness of process implementation [36, 43]. Furthermore, the inclusion of local knowledge raises the quality of decision-making (e.g., [10, 43, 44]). Finally, the *participation* of a diverse range of state and non-state actors also makes multi-level governance systems useful to confront policy issues that *transcend territorial boundaries* [30, 43].

At the same time, multi-level governance can present a series of obstacles to policymaking. Among these, the existing literature has pointed out that the complexity of multi-level systems and their inherent transaction or coordination costs might hamper the effective implementation of environmental policy issues [24]. On the other hand, the empowerment of a more diverse range of actors leads to greater chances of internal contestation and less cohesive policies [30].

According to the “argumentative turn” in political and social sciences [20], ideas and beliefs shape collective action and the allocation of political resources. More specifically, the actor groups involved in the policy process use discourse to influence political negotiations in accordance with their convictions. Hajer ([29], p. 64) defined discourse as “a set of concepts that structure the contributions of a group of participants to a discussion”. For her part, Göpel [25] argued that discourses and narratives constitute the basis of thinking and of economic models, and that system boundaries are redefined in accordance with discursively constructed variables. Hence, discourse conveys useful information to understand actors’ perceptions regarding specific policy issues, or how interactions with other actors influence such thoughts [9]. Discourse analysis [27–29] and discursive institutionalism [47, 48] see political processes as battles for discursive hegemony between competing discourse coalitions [39]. Such dynamics have also shaped the process of European integration over the years [16].

Following these premises, the discourse-coalition approach studies how interrelationships are “produced, reproduced, challenged, and transformed” ([29], p. 63). Hence, actors that share common preferences and are “attracted to a specific [set] of storylines” tend to form coalitions—understood as an “ensemble of storylines, the actors who utter these storylines, and the practices in which this discursive activity is based”—to defend their interests ([28], p. 65). This perspective assumes that the narrative that embeds a particular debate—such as that of air pollution—can shape perceptions of the issue being discussed, for instance, the extent to which actors perceive it as a problem [29]. Hence, linking the discourse-coalition approach to multi-level governance theory will help explain discursive tensions and struggles for power across national, subnational, and supranational levels.

Given the coexistence of various contested interpretations of the causes and remedies for the problems that policymakers of this issue area address, environmental policy is regarded as a socially constructed domain. Each policy actor frames political success from diverging angles, as the considerations (i.e., economic growth, public health) that are assessed for determining the “price” of a good vary in different narratives [25]. Framing and confronting rationalities are both essential aspects of the environmental policymaking process, particularly when it comes to health aspects or individual behavior. Hence, arguments in favor of stricter measures against air pollution are oftentimes refuted by those who point to the effects of such policies on the economy [19]. Traditionally, debates around environmental issues were characterized by the prevalence of language that targeted experts in the field rather than the general public [19]. That being said, the general public has gradually become involved in environmental discussions. Activists such as Greta Thunberg—who recently became a global symbol of the fight against climate change—have contributed to this increase of popular interest in the topic.

In this paper, we combine insights from the discourse-coalition and the multi-level governance approaches to examine the nature and evolution of the debate over the reduction of air-pollution emissions in the EU. We use discourse network analysis to compare the debates at different levels of governance. The next section examines specificities related to the cases that we have selected for this study.

## Air pollution in German cities: the cases of Berlin and Stuttgart

Approved by all United Nations members and EU member states, the 1987 Montreal Protocol on Substances that Deplete the Ozone Layer sped up the fight against air pollution. Five years later, it was followed by the United Nations Framework Convention on Climate Change (UNFCCC), which sought to reduce levels of greenhouse gases and other air pollutants in the atmosphere. In 1997, the Kyoto Protocol established binding obligations for developed countries to reduce their greenhouse gas emissions. This agreement suffered from legitimacy problems, especially after the US—at that time responsible for the highest volume of air pollutant emissions in the world—refused to ratify it, and Canada also withdrew from the arrangement [35]. The signature in 2016 of the Paris Agreement renewed the commitment of most countries in the world to limit global warming. Only six countries did not sign the agreement, which included no obligations for countries to set particular targets and which relies entirely on their willingness to design climate-friendly strategies.

Within the EU context, legislation on air pollution has—in view of its cross-border nature—been strengthened considerably since the signature of the 1979 Convention on Long-Range Transboundary Pollution (LRTAP). This was followed by the approval in 1980 of Directive 80/779/EEC, which limited values for sulfur oxide (SO_2_) and suspended particulate matter (PM). Subsequent directives would regulate emissions of lead (1982 Directive 82/884/EEC, 1999 Directive 1999/30/EC, and 2002 Directive 2002/3/EC); nitrogen dioxide (1985 Directive 85/203/EEC); ozone (1992 Directive 92/72/EEC); carbon dioxide and benzene (2000 Directive 2000/69/EC), and arsenic, cadmium, mercury, nickel, and polycyclic aromatic hydrocarbons (2004 Directive 2004/107/EC). Other legislation has targeted sources of pollution rather than the pollutants themselves. In this regard, the activities of large combustion plants (1988 Directive 88/609/EEC and 2001 Directive 2001/80/EC); positive-ignition engines of motor vehicles (1970 Directive 70/220/EEC), and diesel engines (1987 Directive 88/77/EEC) have attracted special attention. In 1996, the EU implemented Directive 96/61/EC on pollution prevention and control. In that year, the EU member states established a binding commitment to a series of targets for air quality by agreeing on the Ambient Air Quality Framework Directive 96/62/EC [1]. In 2008, the European Parliament and the European Council passed Directive 2008/50/EC, which made maximum caps even stricter for air pollutants, such as SO_2_ and nitrogen oxides (NO). Brussels also obliged member states to design and implement strategies to reduce emissions, in particular to reduce PM [35].

The European Commission formally monitors the implementation of air quality directives. Hence, it may refer those states that exceed the agreed air-pollution caps to the European Court of Justice (ECJ). Such a system puts effective limits on member states’ sovereignty. By 2017, the Commission had taken 12 member states to the ECJ for exceeding the permitted levels of nitrogen dioxide (NO_2_) [17]. In turn, national governments are responsible for transposing the directives adopted by the European Parliament or the European Council, for assessing air quality, for ensuring that air quality data is publicly available, and for taking the necessary measures to prevent air-pollution levels from exceeding the EU standards [24]. National and subnational levels of government can also set stricter limits and deadlines than those prescribed by the EU directives [4]. This limited managerial discretion to implement policies results in outcome variation across the member states [24]. For its part, the European Environment Agency (EEA) has, since 1994, provided a variety of actors—including the Commission, the Parliament, and the member states—with independent scientific advice on the environment. With 32 member states and six cooperating countries, the EEA coordinates several national networks formed by environmental agencies and ministries. It also produces reports concerning topics such as climate change and human health that are targeted at state and non-state actors within and beyond the EU borders.

With a gross domestic product (GDP) of over 3.4 trillion euros in 2019, Germany is the largest economic power in the EU [13]. The automotive industry constitutes a central pillar of economic success in Germany as well as in Europe ([26], p. 147). However, the country is undergoing a profound transformation, as new forms of mobility could threaten its economic strength [26]. In September 2015, the Volkswagen emissions scandal—which became known as “Dieselgate”—damaged the company’s reputation. Volkswagen as well as other companies (e.g., Mercedes-Benz, Audi, and Porsche) were found guilty of manipulating the reported emission values of their vehicles to conform with regulatory standards [45]. Considering that the German economic situation, the presence of a strong automotive industry, and air quality are highly dependent on each other, automotive-related interest groups have an interest in hindering the implementation of stricter measures in this country.

By 2017, Germany had implemented over 130 action plans. Although legal transposition has been a successful experience in Germany, there is an implementation deficit concerning environmental policies [24]. This is partly a consequence of the unwillingness of national and subnational authorities to adopt the necessary administrative and legal changes—with the ensuing political and economic costs—for the effective implementation of EU directives [6]. While the capacities of subnational authorities in Germany have recently expanded, the suboptimal performance of these bodies stems from the vague nature of their competencies and their inability to engage in enforcement activities vis-à-vis higher levels of governance [24]. Moreover, local and regional policymakers in Germany are struggling to implement air quality directives due to insufficient support from the federal government [24].

Within this context, private firms, environmental NGOs, and climate activists have joined policymaking discussions [24]. In this regard, German courts have empowered citizens by granting residents of areas that exceed the established air-pollution caps the right to force authorities to set up emission–reduction plans [12]. In addition, a “greening of politics” has increased the popularity of the German Green Party,^1^ which has enjoyed representation in the German Bundestag since 1983 [15]. In all, a clash of interests between a strong economy that is heavily reliant on the automobile industry and powerful environmental-policy interests shapes the air-pollution debate in Germany.

To study the local level—and following the guidelines of Seawright and Gerring [49]—we have chosen two German cities as our case studies. More specifically, we have selected extreme and influential city cases. In this regard, as a hub of the automotive industries and automotive suppliers (e.g., Porsche, Mercedes-Benz), *Stuttgart* stands out as a city with extremely bad air quality. We have also selected *Berlin* (influential). Besides its status as the capital of Germany, this city hosts local, regional, and national governmental institutions.

The capital of Baden-Württemberg, Stuttgart is home to more than 609,000 residents (as of November 2020). Stuttgart features a distinctive topography that impedes the circulation of air. Situated between two highways and intersected by multilane federal highways, Stuttgart is probably the most important traffic hub in Baden-Württemberg and a relevant economic center in Europe. Indeed, globally active corporations from the automotive and mechanical engineering sectors (e.g., Mercedes-Benz, Porsche, Bosch) have their headquarters in Stuttgart. The gross value added in Stuttgart (i.e., the total value of all goods and services produced in the city, minus so-called “intermediate inputs”) was 47,842 million euros in 2017. This figure corresponds to roughly 11% of Baden-Württemberg’s total production. These circumstances partly explain the bad air quality levels of Stuttgart, despite the fact that almost half of the city area is covered with forests, fields, and parks [22].

With its 3.7 million inhabitants, Berlin is the largest municipality and the capital of Germany, as well as being the most populated city in the EU. Apart from the Spree and Havel rivers, there are numerous lakes and forests in the city area. Berlin stands in stark contrast to Stuttgart in many areas that are relevant to our study. For example, the economy of Berlin is less dependent on industrial revenue, and much less so on the automotive industry. Its inhabitants have fewer cars per capita than those of other major European cities: in 2017, 381.8 motor vehicles were registered per 1000 inhabitants in Berlin—the average figure in Germany in that year was 687 per 1000 inhabitants [3]. Moreover, its public transport system is much better developed than those of other German cities. Berlin also hosts many lobbying organizations, environmental protection organizations (ENGOs), and scientific research organizations, such as the Institute for Advanced Sustainability Studies (IASS). In addition, Berlin’s citizens are highly engaged in pro-environmental activism. For example, in February 2017, civil society organizations brought to the political agenda the *Volksentscheid Fahrrad*, an initiative that received widespread support and resulted in the approval of legislation that increased the safety of bike users. The success of this initiative led other German cities to follow this example.

## Methodology

### Discourse network analysis

In this paper, we examine the nature and evolution of the debate around air-pollution regulation in Europe from a network perspective, which lays emphasis on the interactions between a series of actors [34]. More specifically, we use discourse network analysis (DNA), a methodology that belongs to the family of social network analysis (SNA). SNA is able to produce abundant empirical evidence on diverse matters, such as the evolution of a debate or the handling of a crisis [7]. Such information oftentimes appears in the form of network graphs showing the connections (ties) between a set of actors (nodes), or in tables with numeric indicators regarding network measures, such as centrality^2^ ([58], pp. 169–219 for a detailed explanation).

By combining qualitative content analysis and social network studies, DNA offers a new perspective for tracing the “coevolution of actors and concepts” dynamically and longitudinally ([37], p. 13). To do this, it conceptualizes actor constellations and measures discourse evolution over time ([37], p. 4). Moreover, DNA allows for operationalizing the content and structure of discourses, or in other words, who says what, or which actors are connected by a common narrative [9]. In doing this, DNA might help identify “bridgers”, namely, specific actors who look beyond their closest contacts in order to find new information [11, 23], who control the flow of information between actors [51], or who simply profit from their connections. At the time of writing, the scarcity of multi-level governance studies that have used DNA seems surprising, considering the potential of such a methodology in this field.

In our study, we apply DNA to measure competing discourse coalitions and storylines empirically. In particular, we show one-mode networks of actors that participate in the debate around air pollution, one-mode networks of concepts that such actors use, as well as interactions across and between actors and concepts in two-mode networks. To assess and visualize the development of a particular political discourse—such as the debate over air-pollution control—over time at different political levels, scholars have in the past employed a software tool called Discourse Network Analyzer (see [31, 37]). The use of the Visone visualization software enables a deeper and more intuitive understanding of the content and the dynamic development of the discourse, as well as of the structural constellations of actors and issues [2].

### Data collection and selected time frame

The media provide excellent sources of data for discourse analysis, considering that they accurately represent “discursive struggles around policy problems”, thus allowing for “the empirical representation of ongoing debates, existing conflict lines, and membership in discourse coalitions” ([9], p. 145). Therefore, we selected articles—see Additional file 1: Appendix for a detailed description of the selection process—published by local newspapers of Stuttgart (Stuttgarter Nachrichten and Stuttgarter Zeitung) and Berlin (Der Tagesspiegel and Berliner Zeitung). This choice allows us to cover the German framing of the air-pollution debate in these two cities. To be consistent with this approach, we reviewed the debate in the European institutions by analyzing two German national newspapers (Frankfurter Allgemeine Zeitung and Süddeutsche Zeitung) instead of EU-wide media, such as Politico Europe, Euractiv, or EUObserver. Hence, this methodology helps us capture the most prominent actors and concepts in German newspaper articles that cover this topic.

We linked each statement in the debate to a level of governance. Bearing in mind that certain statements might affect different levels of governance, we assigned statements to specific levels in accordance with the activity of the organization that had made them. In the cases where an organization operated with equal intensity at various levels of governance, we assigned the statement in question to the superior level. For example, the German environmental NGO Deutsche Umwelthilfe (DUH) is active in various German cities and regions; therefore, its statements were coded as national. While this approach may limit the generalizability of our findings, it is systematic, well-founded, and comprehensible.

We selected a time frame ranging from July 2015 (shortly before Dieselgate) to December 2020. We used the EU’s decision to sue Germany for non-compliance with its air-pollution standards on 17th May 2018 as the cutting point of the analysis. Hence, we were able to compare the evolution of the different networks before and after this event. We selected newspaper articles using the keywords “Luftverschmutzung” (air pollution), or “Luftqualität” (air quality) in combination with “Stuttgart”, “Berlin”, or “EU”. We examined 299 documents in total, randomly selecting 100 documents covering the debate in Stuttgart, 100 covering the debate in Berlin, and 99 corresponding to the debate in the EU institutions. Random selection of newspaper articles is a common procedure in DNA studies, as it minimizes manual coding efforts without compromising the quality of the data. In this regard, we know from Kammerer and Ingold [33] and Nagel and Satoh [42]—who used samples ranging from 10 to 25% of the originally selected articles—that, due to a saturation effect, the analysis of coded articles does not throw new insights beyond a certain threshold of articles. To make sure that we did not miss any important concepts or actors, we randomly scanned and reviewed the remaining uncoded articles.^3^ We also conducted in-depth interviews with experts serving in organizations involved in the debates in Berlin (eight) and Stuttgart (nine) to verify the quality of the discourse data. In these interviews, we enquired about the relevance of different actors in the debate to confirm that our analysis had not overlooked any important organizations. Due to space limitations and the analytical focus of the paper, we only used the insights from these interviews for data verification purposes.

Within the selected newspaper articles, we manually coded information about the name of every actor, their organization, statement category, and agreement or disagreement with the concept in question for every statement (direct or indirect speech) in the text. We categorized the statements using a combination of deductive coding processes (predefined categories) and inductive coding processes (expanded during the process) (for more details, see [41]). We have included in Additional file 1: Appendix a more detailed description of the criteria we used to categorize actors according to their type of organization and level of governance.

## Analysis and results

**Fig. 1:**
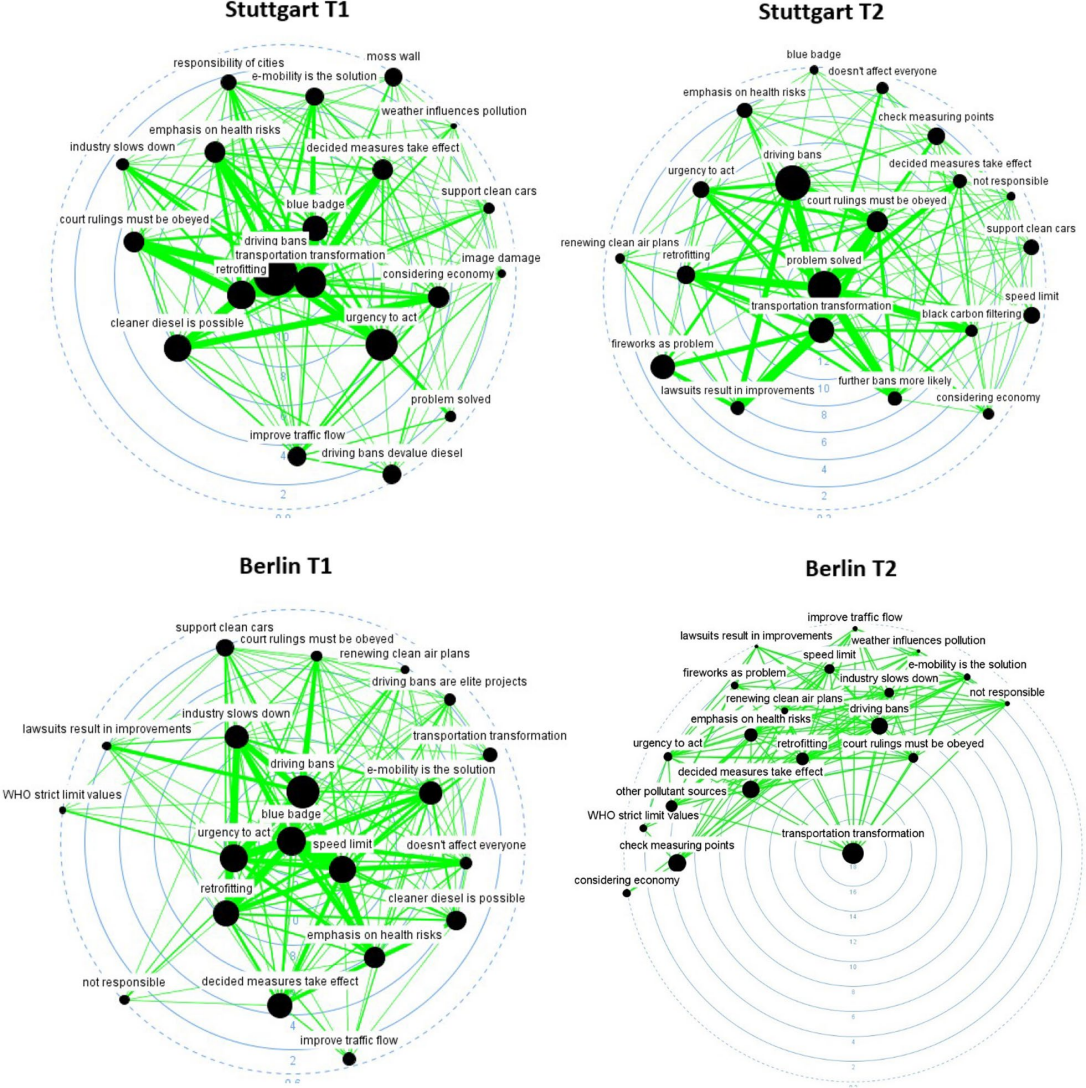

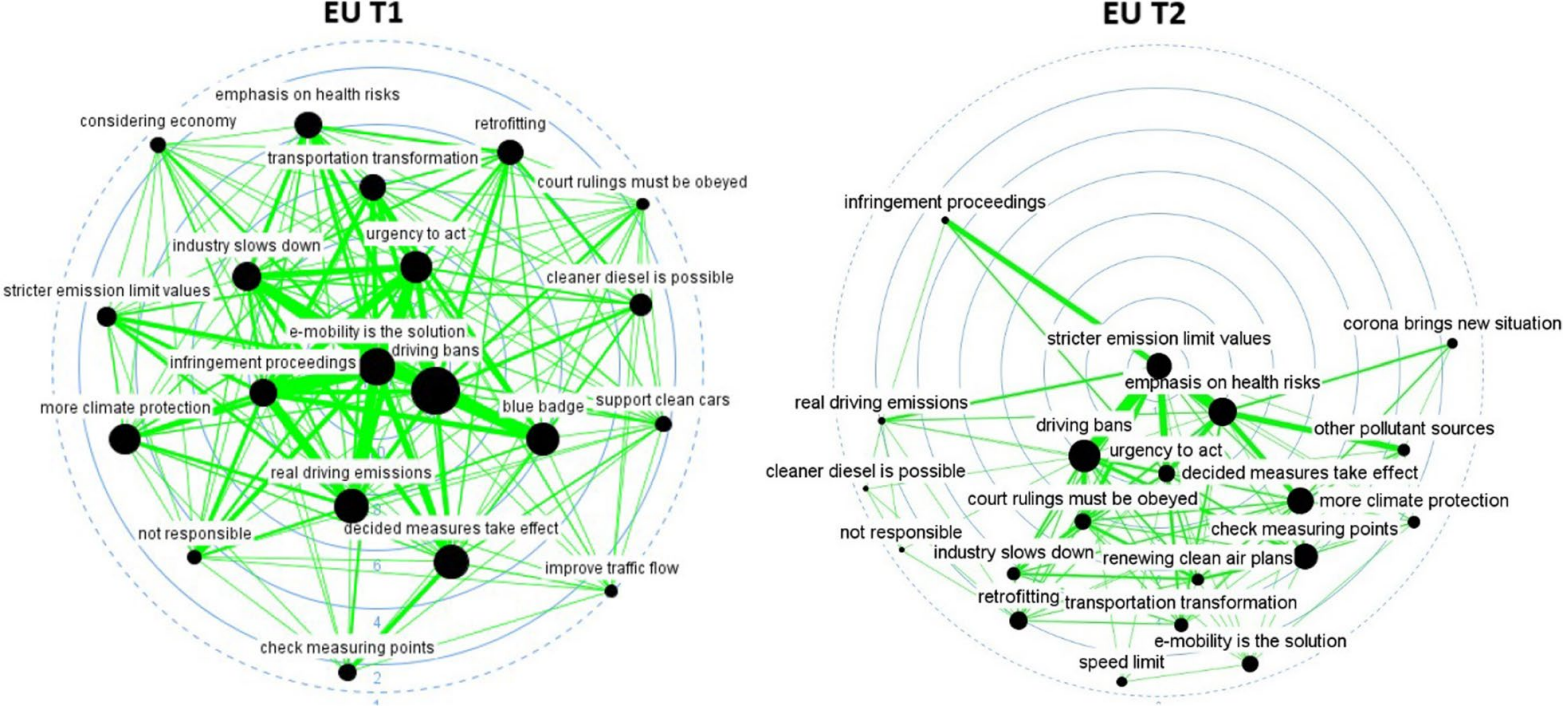
Radial layouts representing debates in Stuttgart (top), Berlin (center), and the EU (bottom). Figures to the left correspond to T1 (before the Commission sued Germany); those to the right correspond to T2 (after the Commission sued Germany)

In this section, we analyze a series of discourse networks. For this analysis, we first refer to radial layouts, where concepts are represented as nodes that are connected through actors sharing an opinion on them (Fig. 1). In these kinds of graphs, large node sizes indicate topics that are frequently discussed in newspapers, whereas edge weights (the thickness of the ties) indicate the number of actors that share the same opinion on two given concepts. Finally, degree centrality values—represented by small numbers near each of the concentric circles—show the number of connections between topics in the debate. In other words, actors raise topics with a higher degree centrality more often in combination with other issues.

**Fig. 2:**
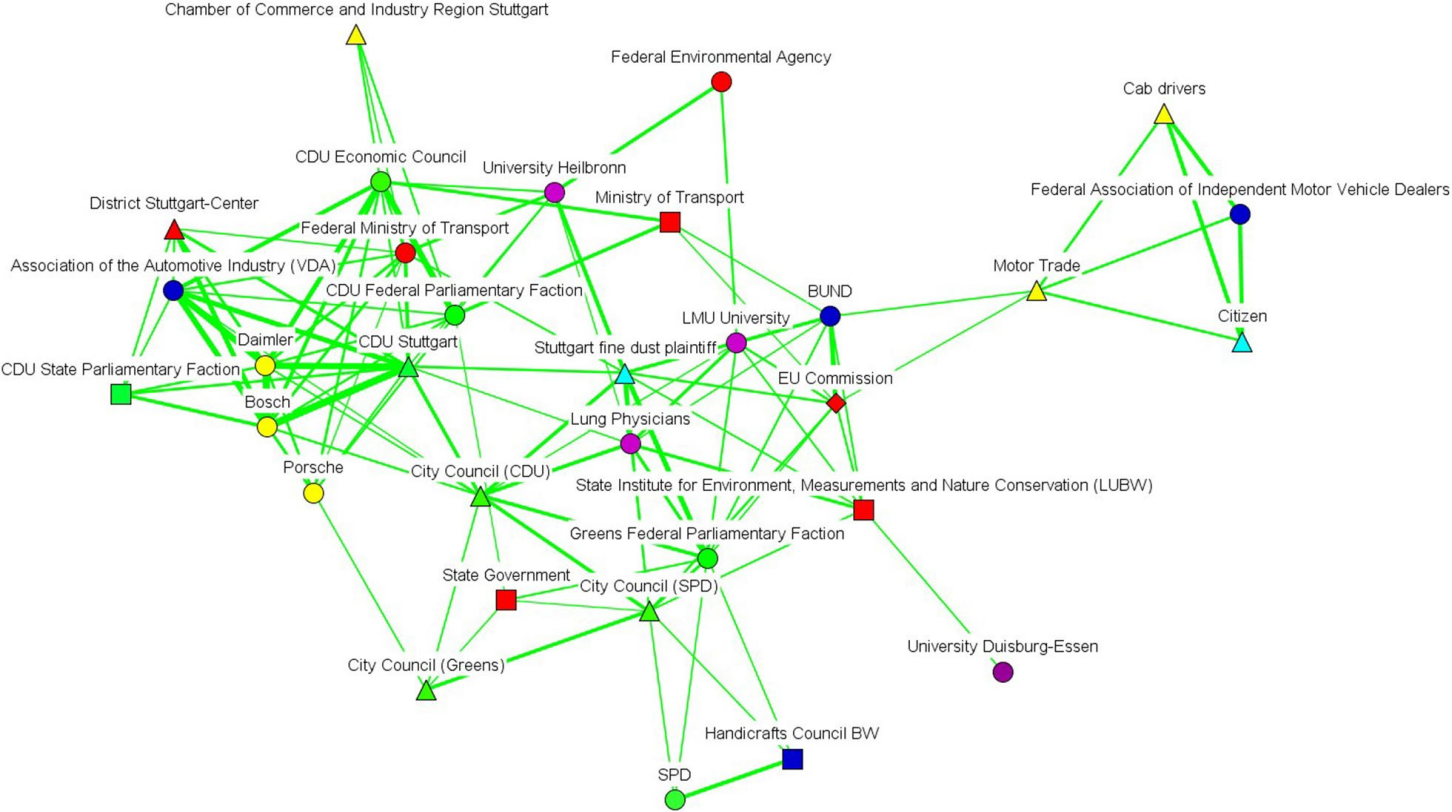
Discourse network in Stuttgart (T1), threshold > 0.6. Source: own elaboration. The color of each node indicates the different organization types. Thus, government actors appear in red; NGOs in navy blue; economic actors in yellow; grassroots initiatives in light blue; political organizations in green; public-sector economic actors in gray; international organizations in white, and scientific bodies in purple. For their part, node shapes reveal the affiliation of each actor to a particular level of government: hence, nodes corresponding to local and regional actors appear as triangles; state (Land) actors as square; national-level organizations as circles, and supranational bodies as diamonds

**Fig. 3:**
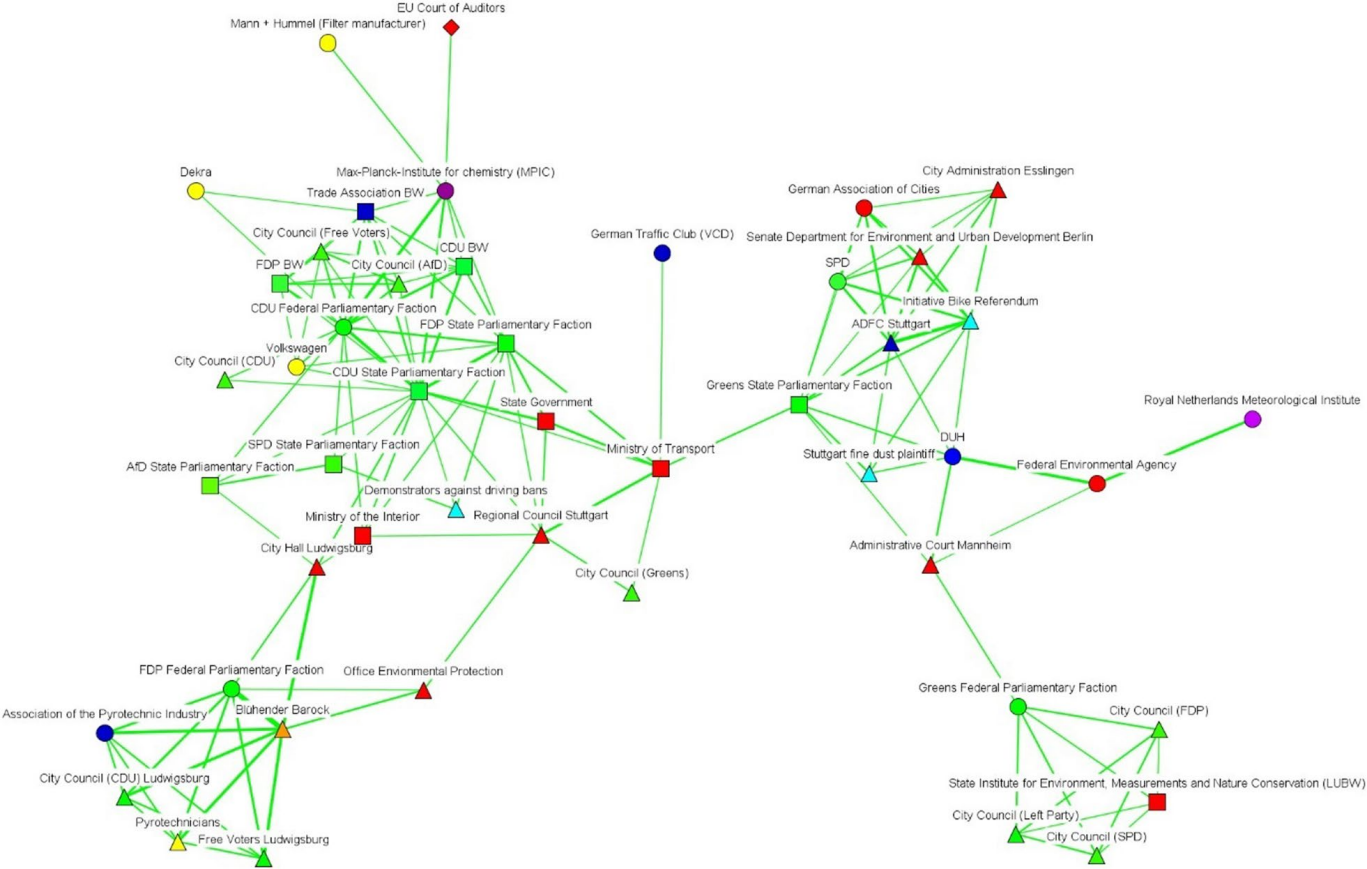
Discourse network in Stuttgart (T2), threshold > 0.316. (Source: own elaboration. See Fig. 2)

**Fig. 4:**
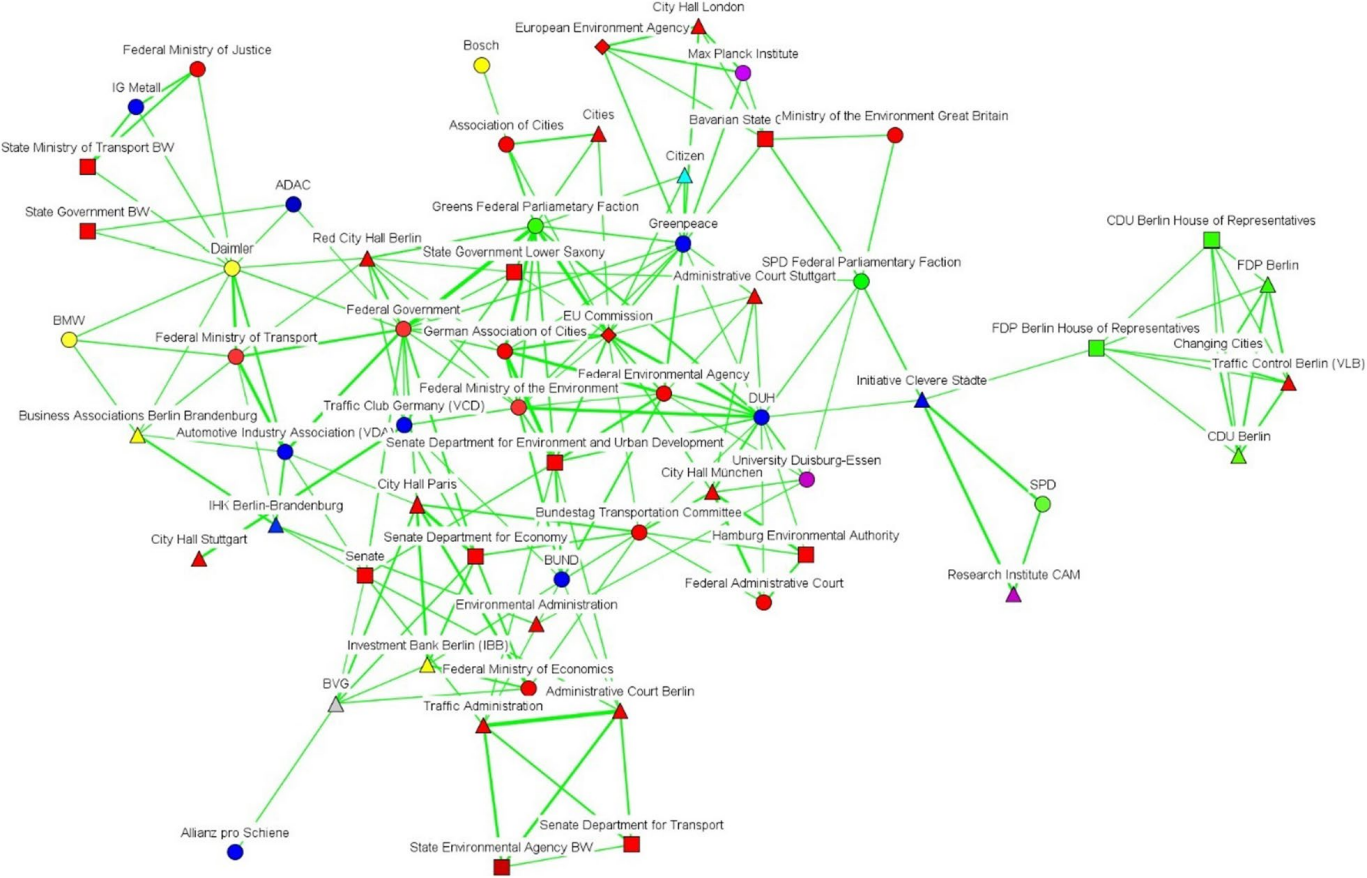
Discourse network in Berlin (T1), threshold > 0.522 (Source: own elaboration. See Fig. 2)

**Fig. 5:**
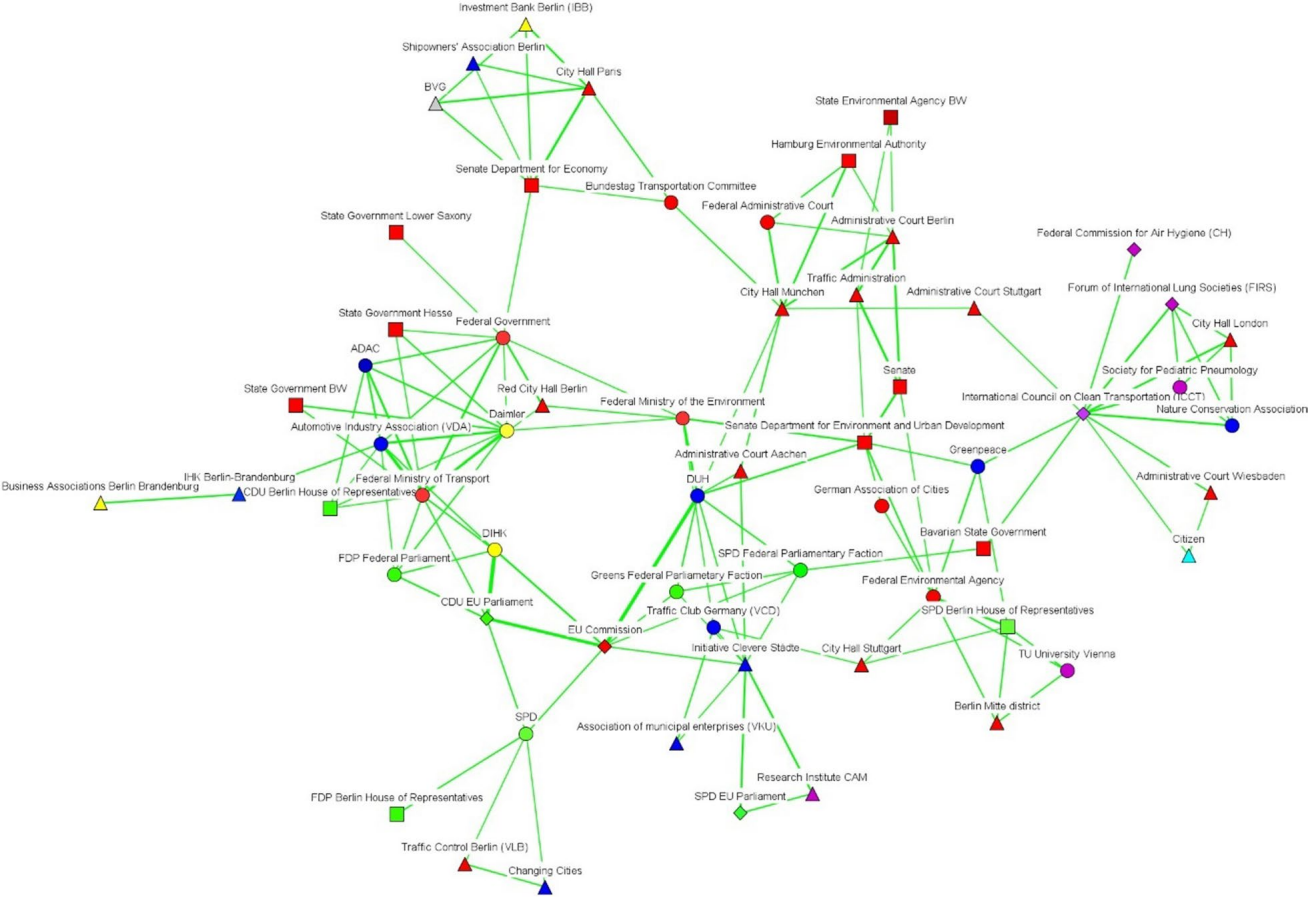
Discourse network in Berlin (T2), threshold > 0.759 (Source: own elaboration. See Fig. 2)

**Fig. 6:**
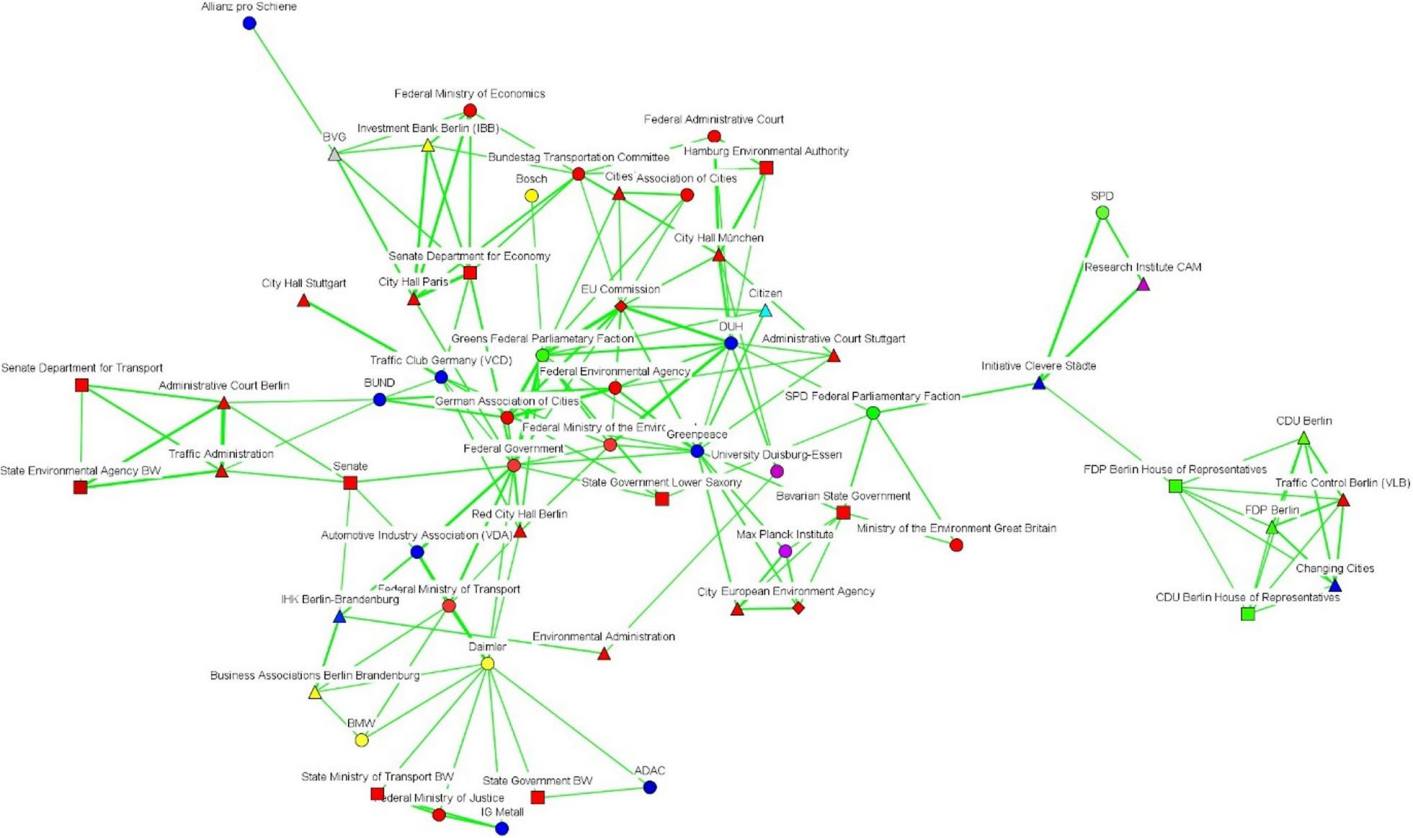
Discourse network in the EU institutions (T1), threshold > 0.562 (Source: own elaboration. See Fig. 2)

**Fig. 7:**
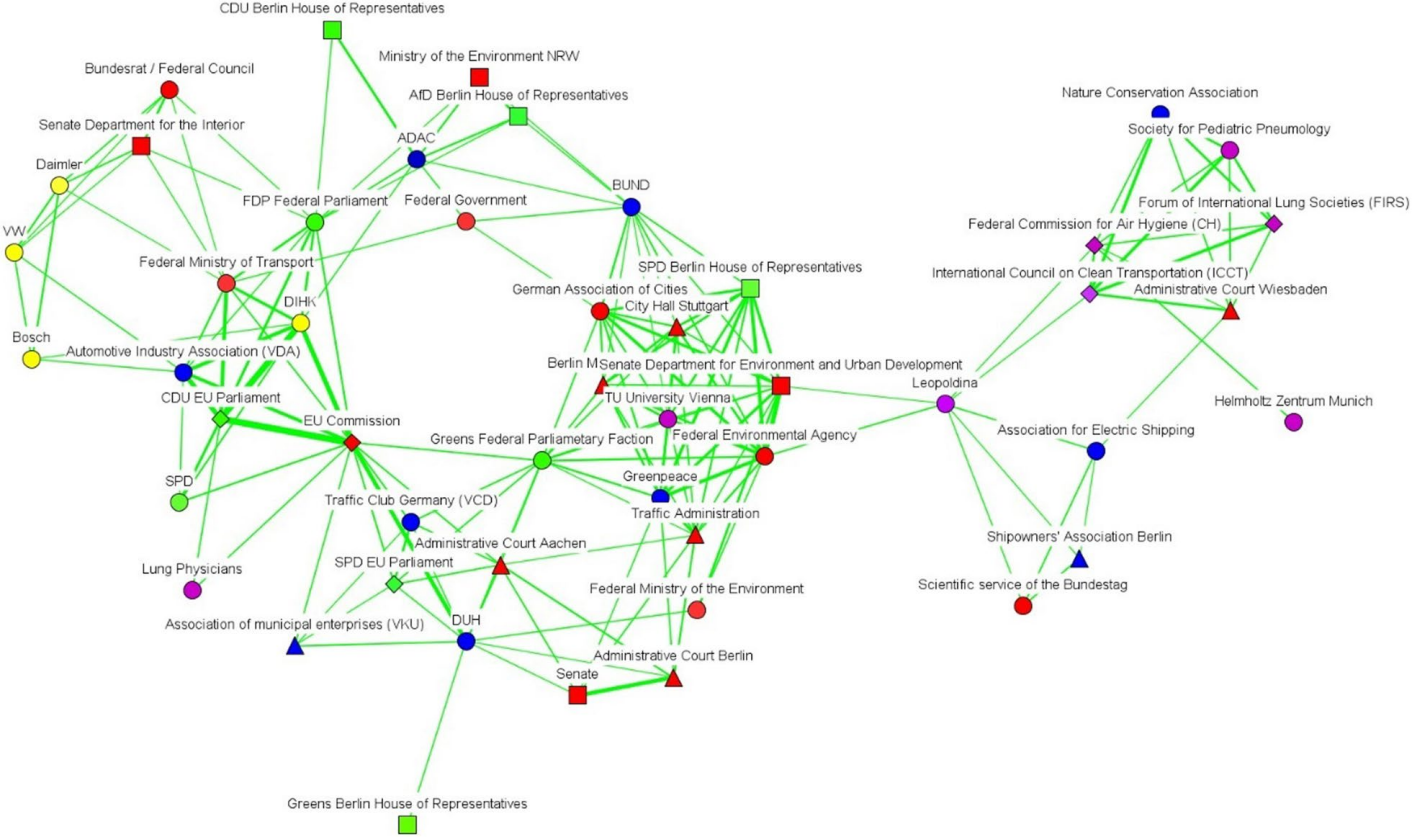
Discourse network in the EU institutions (T2), threshold > 0.453 (Source: own elaboration. See Fig. 2)

We have also incorporated subtract one-mode networks (Figs. 2, 3, 4, 5, 6 and 7), where nodes represent actors, and ties show the actors’ conceptual alignment: in these networks, we calculated edge weights by subtracting the number of conceptual agreements from the number of disagreements. Hence, two actors are connected to each other if they agree about more concepts than they disagree about. These networks are useful to examine polarized debates, such as that of air pollution [38]. In addition, we include two-mode networks that show the position of each discourse coalition concerning the most important issues in the debate (see the Additional file 1: Appendix).

Finally, Tables 1, 2, 3, 4, 5 and 6 present the 15 organizations with the highest degree centrality and frequency scores within each subtract one-mode network. Both degree centrality and frequency are helpful indicators to identify the most prominent actors within each network. More specifically, actors with higher degree centrality values in these tables are better connected through discursive congruence with other actors in the debate. Concerning frequency, we used a threshold of at least 4 to get a clearer interpretation of the graphs by removing those actors that were mentioned only a few times in the debate. For the visualizations, we used a step-by-step procedure described in Nagel [41] to calculate specific threshold values. We normalized such scores on a scale from 0 to 1. At this point, we must mention that the different number of nodes in the examined networks and the non-uniform application of tie thresholds across figures made it impossible for us to analyze network metrics, such as density^4^ in a comparative fashion. For further information regarding this procedure, see the Additional file 1: Appendix.

**Table 1:** Degree centrality scores in the Berlin (T1) network. Frequency > 4

Name of the organization	Type of organization	Level of governance	Frequency	Degree centrality score
Greens Federal Parliamentary Faction	Political	National	15	8.357
Federal Government	Gov/Admin	National	18	7.816
German Association of Cities	Gov/Admin	National	13	7.145
European Commission	Gov/Admin	Supranational	8	6.81
Senate Department for Environment and Urban Development	Gov/Admin	State	46	6.604
Federal Ministry of the Environment	Gov/Admin	National	25	5.898
DUH	NGO	National	41	5.775
Federal Environmental Agency	Gov/Admin	National	7	4.88
Greenpeace	NGO	National	6	4.563
Red City Hall Berlin	Gov/Admin	Local	8	4.563
Federal Ministry of Transport	Gov/Admin	National	13	4.557
Automotive Industry Association (VDA)	NGO	National	21	4.553
Daimler	Economy	National	5	4.419
Senate	Gov/Admin	State	9	3.707
BUND	NGO	National	5	3.438

**Table 2:** Degree centrality scores in the Berlin (T2) network. Frequency > 4

Name of the organization	Type of organization	Level of governance	Frequency	Degree centrality score
European Commission	Gov/Admin	Supranational	10	12.18
CDU EU Parliament	Political	Supranational	4	12.157
DIHK	Economy	National	5	11.514
Federal Ministry of Transport	Gov/Admin	National	11	7.952
Automotive Industry Association (VDA)	NGO	National	4	7.604
Senate Department for Environment and Urban Development	Gov/Admin	State	39	6.44
Federal Environmental Agency	Gov/Admin	National	5	6.016
German Association of Cities	Gov/Admin	National	4	5.578
DUH	NGO	National	27	5.559
ADAC	NGO	National	9	3.878
Federal Ministry of the Environment	Gov/Admin	National	8	3.79
CDU Berlin House of Representatives	Political	State	8	3.525
Leopoldina	Scientific	National	14	3.256
Federal Government	Gov/Admin	National	4	3.226
Lung Physicians	Scientific	National	9	3.067

**Table 3:** Degree centrality scores in the Stuttgart (T1) network. Frequency > 4

Name of the organization	Type of organization	Level of governance	Frequency	Degree centrality score
City Hall Ludwigsburg	Gov/Admin	Local	11	5.061
Daimler	Economy	National	8	4.886
CDU Stuttgart	Political	Local	15	4.857
European Commission	Gov/Admin	Supranational	10	4.814
City Council (CDU)	Political	Local	11	4.768
Bosch	Economy	National	13	4.68
Association of the Automotive Industry (VDA)	NGO	National	15	4.404
Stuttgart Fine Dust Plaintiffs	Grassroots initiative	Local	4	4.394
City Council (SPD)	Political	Local	7	4.343
City Council (Greens)	Political	Local	25	3.783
Greens Federal Parliamentary Faction	Political	National	6	3.742
DUH	NGO	National	6	3.701
BUND	NGO	National	4	3.437
Federal Ministry of Transport	Gov/Admin	National	13	3.336
CDU Economic Council	Political	National	11	3.208

**Table 4:** Degree centrality scores in the Stuttgart (T2) network. Frequency > 4

Name of the organization	Type of organization	Level of governance	Frequency	Degree centrality score
CDU State Parliamentary Faction	Political	State	17	10.458
FDP State Parliamentary Faction	Political	State	10	9.018
CDU Federal Parliamentary Faction	Political	National	5	8.876
Ministry of Transport	Gov/Admin	National	41	7.835
State Government	Gov/Admin	State	24	6.937
Max–Planck-Institute for Chemistry (MPIC)	Scientific	National	6	6.461
CDU BW	Political	State	5	5.981
City Hall Ludwigsburg	Gov/Admin	Local	7	5.7
Trade Association BW	NGO	State	5	5.555
Regional Council Stuttgart	Gov/Admin	Local	13	5.482
Office of Environmental Protection	Gov/Admin	Local	4	4.185
Demonstrators against Driving Bans	Grassroots initiative	Local	9	4.062
Dekra	Economy	National	5	3.984
Greens State Parliamentary Faction	Political	State	9	3.821
City Council (Greens)	Political	Local	6	2.494

**Table 5:** Degree centrality scores in the EU (T1) network. Frequency > 4

Name of the organization	Type of organization	Level of governance	Frequency	Degree centrality score
DUH	NGO	National	19	9.229
Greens Federal Parliamentary Faction	Political	National	8	8.469
Ministry of Transport BW	Gov/Admin	State	16	8.382
European Commission	Gov/Admin	Supranational	42	7.258
Federal Ministry for the Environment	Gov/Admin	National	27	7.139
State Government BW	Gov/Admin	State	15	6.406
BUND	NGO	National	12	6.14
German Association of Cities	Gov/Admin	National	8	5.512
Greenpeace	NGO	National	4	5.165
Regional Government Brussels	Gov/Admin	State	4	4.979
VW	Economy	National	11	4.664
Daimler	Economy	National	9	4.423
Federal Ministry of Economics	Gov/Admin	National	5	3.783
Environmental Ministry France	Gov/Admin	National	6	3.625
Federal Environmental Agency	Gov/Admin	National	4	3.445

**Table 6:** Degree centrality scores in the EU (T2) network. Frequency > 4

Name of the organization	Type of organization	Level of governance	Frequency	Degree centrality score
European Commission	Gov/Admin	Supranational	16	9.074
European Environment Agency (EEA)	Gov/Admin	Supranational	12	7.912
Leopoldina	Scientific	National	4	7.784
Federal Government	Gov/Admin	National	6	7.021
CDU BW	Political	State	6	7.004
State Government Hesse	Gov/Admin	State	6	6.291
International Council on Clean Transportation (ICCT)	Scientific	Supranational	4	6.153
University Düsseldorf	Scientific	Local	4	6.115
Federal Environmental Agency	Gov/Admin	National	11	5.656
DUH	NGO	National	18	4.982
Federal Ministry of Transport	Gov/Admin	National	19	4.548
European Court of Justice	Gov/Admin	Supranational	6	4.384
State Government BW	Gov/Admin	State	10	4.225
Administrative Court Mannheim	Gov/Admin	Local	4	4.053
Ministry of Transport BW	Gov/Admin	State	8	3.876

### Analyzing air pollution from a multi-level governance perspective

To better illustrate the background of the two cities, we now outline the most prominent narratives and policy measures adopted in each case. In both cities, public authorities understood the need for air quality to improve, and they introduced policy measures for this purpose. Therefore, the starting situation in both Stuttgart and Berlin is very similar. Both cities sought to reduce air pollution and CO_2_ emissions for environmental and health reasons to comply with the EU values. In the first time frame (T1, 7/2015 to 5/2018), policy measures were discussed in Stuttgart and Berlin. In the second time frame (T2, 5/2018 to 12/2020), policy measures were defined. Each city published an official clean-air plan in 2019.

These documents differ considerably from one another, though. As the *Clean Air Action Plan* from November 2019 [46] shows, creative measures were taken in Stuttgart to improve air quality without reducing the number of vehicles emitting pollutants. Examples of these include the construction of air filter columns, the application of innovative road surfaces, painting facades with photocatalytic facade paint, and software updates for diesel vehicles. In contrast, the *Air Quality Plan for Berlin* 2019 [50] emphasizes the improvement and increase of public transport systems. Berlin’s approach also highlights the need for cycling and pedestrian plans. Furthermore, it calls for replacing public transportation vehicles with e-buses, e-cars, and other emission-free vehicles.

We now turn to the public debates in each city. Discussions in Stuttgart focused on the dominance of cars in the city and on parking lots. The reachability of restaurants and shops by car was emphasized too. Since many Stuttgart citizens work in the local automotive industry, the narrative associates cars with the city’s economic success. Indeed, the mayor of Stuttgart recently emphasized in a newspaper article that “the city center must be reached by car” [52]. In the case of Berlin, the local narrative emphasizes air pollution and emissions reductions. For its part, the concept of environmental justice is deeply rooted in public policy and in the administration of the city. During the COVID-19 pandemic, the debate in Berlin highlighted the installation of temporary bike lanes and areas free from traffic (i.e., *Kiezblocks*). The local narrative also reflects pride in the public transport system. For instance, BVB—the main public transport company in Berlin—is very popular among Berlin citizens. With these different insights in mind, we can now delve deeper into the analysis.

Figure 1 shows that the driving bans imposed on cars that did not comply with air pollutant emission standards, and the transformation of transport, remained prominent topics in all networks during these years. A pro-environmental discourse emphasizing aspects such as the urgent need for tightening measures against air pollution, or the health risks of air pollution, dominated discussions in the EU network (see the Additional file 1: Appendix for an explanation of each concept). Reflections on the effects of the measures on the overall economy seem to be much less salient in the EU network than in the Stuttgart network. In the two German cities, the debate evolved through the creation of consistent storylines that influenced public opinion in different ways. For example, claims that air pollution had already been solved and did not require urgent action became more prominent in Stuttgart after the Commission took Germany to court. At that time, Stuttgart remained the second most polluted city in Germany [8] even after the dubious installation of air filtration systems right next to public measuring stations [21]. In Berlin, the promotion of alternative means of transportation and reflections that the measures adopted to tackle air pollution had to come into force became the center of the debate after the Commission sued Germany. This circumstance evidences the differences in framing between the cities. The debate in the EU network also differs greatly from those in both German cities concerning the salience of concepts. In any case, the debate in the EU network appears to be better aligned with the topics discussed in Berlin than with the pro-economic-interest arguments that dominated discussions in Stuttgart.

Concerning actor prominence in each network, Tables 1, 2, 3, 4, 5 and 6 reinforce our impression that the debates in the EU institutions and in Berlin shared a number of patterns, whereas discussions in Stuttgart tended to follow a different logic. Indeed, the high degree centrality scores of political parties in Stuttgart evidence a higher politicization of the debate compared with the Berlin and EU networks, where governmental institutions tended to lead discussions. On the other hand, supranational and national organizations tended to drive the debate both in Berlin and in the EU institutions. In contrast, local and state actors tended to drive the debate in Stuttgart. Indeed, actors such as the European Commission were more prominent in the Berlin and the EU networks than in the debate in Stuttgart.

After turning our attention to the subtract one-mode networks, we can see that lobbies such as the Association of the Automotive Industry (VDA), economic firms (e.g., Daimler, Bosch), and political bodies (e.g., the CDU Economic Council) seemed to form part of a large pro-economic-interest coalition in Stuttgart before 2018 (see Fig. 2). A smaller coalition—including bodies such as the Government of Baden-Württemberg, the German non-governmental organization BUND, and the European Commission—defended pro-environmental arguments. In both time frames, governmental organizations at the state level, such as the State Ministry of Transportation or the state (*Land*) government, seemed to act as bridges between the discourse coalitions in favor of reducing air pollution and the larger automotive-protecting discourse coalition. After 2018, this larger discourse coalition was led by the German political parties CDU and FDP, trade associations, and the grassroots movements that channeled protests against driving bans (see Fig. 3).

In Berlin, there was an increase over time in the number of discourse coalitions (see Figs. 4 and 5), with governmental bodies constituting a large share of the actors in both networks. After the Commission took Germany to court, governmental actors belonging to different levels of governance positioned themselves as bridges between the different discourse coalitions. For their part, Figs. 6 and 7 show that scientific organizations and lobbies had an important weight in the EU network. After Germany was sued for non-compliance of EU air-pollution standards, Leopoldina, the Federal Environmental Agency, and the Senate Department for the Environment and Urban Development, bridged a pro-environmental discourse coalition, which mainly included scientific organizations, with the rest of the network. All three cases show a tendency for governmental actors to act as bridges between discourse coalitions.

## Conclusions and discussion

We have analyzed the nature and evolution of the debate on air pollution by linking the multi-level governance and discourse-coalition frameworks. Our DNA data allow us to identify different discursive arenas characterized by conflicts within and across levels, where different actors pushed for advancing their normative agendas. More specifically, a great scientific emphasis on air-pollution health risks and the relatively small presence of private interests characterized the debate in the EU network. In contrast, the two German cities reacted in different ways to the EU decision to take Germany to court due to its non-compliance with the standards for air pollution set in Brussels.

Both cities face the problem of air pollution and thus implemented a plan in 2019 to reduce emissions. However, these clean-air plans differed dramatically from one another. While Berlin implemented several measures to reduce the number of vehicles emitting pollutants in the city, Stuttgart implemented creative measures that did not center around the reduction of vehicles that emitted pollutants. In this regard, our longitudinal DNA shows that debates around the approval of new measures against air pollution align with these diverging trajectories and became much more prominent over time in Berlin than in Stuttgart, where actors increasingly referred to air pollution as a resolved matter. The relative prominence of certain actors (e.g., non-governmental interest groups, private firms, governmental institutions) within the dominant discourse coalitions in each city helps make sense of the extent to which debates at the local level align with the arguments put forward by the EU institutions. This variation evidences the ability of local actors to shape the air-pollution debate within Europe.

If we compare the evolution of air-pollution values in both cities, we can see that particulate matter PM10 and PM2.5 values decreased to a greater extent in Berlin than in Stuttgart [54, 55]. This could hint that policy measures in Berlin have been more effective. However, we cannot assume a direct causal link here, as other factors might come into play. Unfortunately, carrying out the required analysis for confirming the existence of such causal links is beyond the scope of this paper.

The existing literature highlights that policy implementation is often problematic in multi-level governance settings due to local inadequacies [24, 56]. By mapping local debates in two cities, we shed light on possible reasons behind such implementation problems. In this regard, the debate in Berlin resembled that in the EU institutions. It seems that the existence of an institutional link through international NGOs, as well as governmental organizations at the national and supranational levels, led to a non-politicized exchange of ideas and concepts. Lacking such a link, and subject to the strong influence of the car industry as well as of local and state actors, Stuttgart framed this debate in more politicized terms. We have also shown the tendency for governmental bodies to act as bridges between discourse coalitions. Hence, our paper might contribute to multi-level governance studies by showing the importance of debates at the local level and their linkages to discussions at higher levels for the implementation of EU policies. In this regard, the ability of supranational and national bodies to align their discourse with those of local organizations might facilitate a common understanding and the successful implementation of EU policies.

Policymakers aiming for the improvement of air quality at the local level may want to consider engaging actively with this debate by reinforcing the arguments of pro-environmental coalitions. They may also need to make a special effort to ensure that the messages conveyed to national and supranational bodies are also effectively transmitted to lower levels of governance. Central to this purpose is the need to explain complex messages to the citizens to increase their commitment. We also argue that policymakers should face the problem of air pollution and take scientific findings and recommendations, such as the WHO Air Quality Guidelines (AQGs) [59], seriously. Accepting these unpopular truths would lead to more effective measures.

Before concluding, we must not forget that our selection of newspapers generates a selection bias in the debates that we have analyzed in this paper—especially the discussions in the EU institutions—in the sense that they reflect the German framing of these issues. Moreover, our reliance on media articles might have led us to underestimate the real influence of the automobile industry and, given that lobbying practices do not always appear in the media, to capture the full picture of the debate inaccurately. For these reasons, additional qualitative research would help triangulate our sources, confirm our preliminary findings, and shed further light on issues that this study did not cover in depth due to space limitations. Such issues include an in-depth analysis of the mechanisms behind the non-uniform implementation of EU legislation at the local level, as well as the role of governmental bodies as bridges between levels of governance. Future papers could go beyond the focus of this paper on the local and supranational levels of governance during policy implementation by exploring their interaction with the less-explored national and regional administrations.

## Supplementary Information

**Additional file 1:** Appendix “Coding scheme, data collection and coding process, categorization of actors, and additional visualization”.

## Data Availability

the data sets used and/or analyzed during the current study are available from the corresponding author on reasonable request.

## Footnotes

1 Indeed, the German Green Party received 20.5% of the votes at the 2019 election for the European Parliament [18].

2 According to Wasserman and Faust (58, p. 173), central nodes conduct extensive exchanges with many other actors.

3 For a detailed explanation of the coding procedures (including inter-coder reliability tests), see Additional file 1: Appendix.

4 Moreover, variations in density across networks or across levels of governance might to some extent stem from a bias in the sample of newspapers examined for this study. Hence, the higher number of ties at the local or national levels does not necessarily mean that the debate was more intense at those levels.
