# Supplementary material for: Analyzing multi-level governance dynamics from a discourse network perspective: the debate over air pollution regulation in Germany
Source: Environ Sci Eur. 2022 Jul 14;34(1):62. doi: 10.1186/s12302-022-00640-0 (PMC9281339; doi:10.1186/s12302-022-00640-0)
Supplement: Supplementary file 1 — Additional file 1: Appendix “Coding scheme, data collection and coding process, categorization of actors, and additional visualization”. [file 12302_2022_640_MOESM1_ESM.docx]

**Appendix**

**1) Coding Scheme**

| Concept | Meaning |
| --- | --- |
| autonomous driving | Autonomous driving is the future. Mobility will be different in the future. |
| black carbon filtering | Fine dust filters are a means to clean the air. |
| blue badge | Introduction of a blue sticker that would allow cars to be marked according to their pollutant emissions and would replace driving zones. |
| cars pollute more real driving emissions | Tests for emission standards are biased, as they do not measure consumption in actual road traffic. Hence, revisions of these tests are considered necessary. |
| check measuring points | Measuring points are closer to the streets in Germany than in other countries. Therefore, values are not comparable. Either measuring points must be revised, or values must be limited. The deadline for compliance with the values must be extended. |
| cleaner diesel is possible | Old diesel engines are the only problem; future engines will be clean. Emphasis on the potential for a better CO_2_ balance in diesel engines. |
| corona brings new situation | The effects of the COVID-19 pandemic have created a new scenario regarding air-pollution emissions. |
| court rulings must be obeyed | Reference to the need to obey court orders requiring strict air-pollution control measures. Rejection means that decisions should be challenged if possible. |
| decided measures take effect | Rejection means that further measures against air pollution are demanded because the old ones are insufficient. |
| driving bans | Agreement means acknowledging that driving bans can make a positive contribution; rejection means emphasizing the economic damage and the harm caused to consumers, as well as the fact that other measures than driving bans are preferable. |
| driving bans are elite projects | Driving bans are directed against the "common people" and are decided by an elite. |
| driving bans lower the value of diesel | Driving bans lead to a sudden loss in the value of diesel cars and thus owners are harmed and deprived of their assets. |
| DUH is not non-profit | The non-profit character of the DUH should be withdrawn. |
| e-mobility is the solution | New, electric vehicles will solve the problems posed by air pollution. |
| emphasis on health risks | The consequences for health in the absence of measures against air pollution are emphasized. |
| fireworks as problem | Fireworks or at least private fireworks on New Year's Eve should be banned; the negative effects of fireworks are problematized. |
| free public transport | Free public transport is demanded (agreement) or rejected (disagreement). |
| further bans more likely | Actors that agree with this statement argue that driving bans are a sign that car use will not be allowed in the future. The argument suggests that more bans may follow to protect health and the environment. |
| image damage | Increasing air pollutant emission values will damage the image of the city; decreasing levels will improve its image again. |
| improve traffic flow | Congestion prevention strategies are suggested as means to reduce pollution. |
| industry slows down | The automotive industry and its suppliers are criticized for not being very innovative when it comes to developing new technologies. |
| infringement proceedings | EU infringement proceedings against Germany are demanded (agreement) or rejected (disagreement). |
| lawsuits result in improvements | Lawsuits by themselves lead sued cities to strive for better values. |
| more climate protection | More ambitious climate protection targets and specifications for the automotive industry are being demanded (agreement) or rejected (disagreement). |
| moss wall | The pilot project “Moss Wall in Cannstatter Straße” is supported and high hopes are placed on it. In the case of rejection, the experimental character of the project is brought to the fore, or rejected altogether. |
| not responsible | Actors describe themselves as not responsible for the problem. |
| only parts involved | Only small streets or residential areas have high air pollutant emission levels. |
| other pollutant sources | One (or more) pollutant source(s) besides road traffic is identified as a problem (e.g., agriculture, shipping, aviation). |
| problem solved | Falling air pollutant emission values show that the problem has already been solved, or is at least about to be solved. |
| renewing clean air plans | A revised, stricter version of the air pollution control plans is demanded (agreement) or rejected (disagreement). |
| responsibility of cities | Each city/municipality should decide for itself how to keep its air clean. Rejection means that nationwide rules are demanded. |
| retrofitting | Hardware or software upgrades are required, or seen as solutions (agreement). Neither hardware nor software updates solve the problem (disagreement). |
| social dimension | The social dimension of driving bans (i.e., their effects on the "common people") is emphasized. |
| speed limit | Calls for lower speed limits on highways or in cities. |
| stricter emission-limit values | Calls for tighter emission limits in line with the stricter WHO standards. |
| support clean car production | Manufacturers need to be incentivized to produce cleaner vehicles in the future; consumers need to be incentivized to buy them. |
| taking the economy into consideration | Measures must take the position of car manufacturers into account. |
| tax concessions for diesel | Tax privileges for diesel fuel and diesel vehicles are defended (agreement) or rejected (disagreement). |
| transportation transformation | Alternative means of transportation (e.g., public transport or bicycles) are incentivized. |
| urgency to act | The need for urgent action is highlighted (agreement) or rejected (disagreement). |
| weather decisive | The weather is a decisive factor in exceeding or falling below the air pollutant emission-limit values. |
| WHO limit values | The stricter WHO air pollutant emission-limit values must (agreement) or must not (disagreement) be taken as a benchmark. |

**2) Data Collection and Coding Process**

| **Selection of data source** | **Keywords and selection process** |
| --- | --- |
| **Berlin**  Newspapers: Der Tagesspiegel and Berliner Zeitung  Number of copies sold and coverage: 364,000 / 0.30 million readers (Der Tagesspiegel)^[[1]](#footnote-1)^; 85,000 / 0.28 million readers (Berliner Zeitung)^[[2]](#footnote-2)^  Political orientation: liberal | “Luftverschmutzung” (air pollution) or “Luftqualität” (air quality) in combination with “Berlin”  Total selected articles: 406  Random sample analyzed: 100  Number of statements: 617  Number of individuals: 191  Number of concepts: 30 |
| **Stuttgart**  Newspapers: Stuttgarter Zeitung and Stuttgarter Nachrichten  Number of copies sold and coverage: 180,000 / 0.49 million readers (Stuttgarter Zeitung and Stuttgarter Nachrichten)  Political orientation: liberal | “Luftverschmutzung” (air pollution) or “Luftqualität” (air quality) in combination with “Stuttgart”  Total selected articles: 989^[[3]](#footnote-3)^  Random sample analyzed: 100  Number of statements: 686  Number of individuals: 189  Number of concepts: 31 |
| **EU level**  Newspapers: Frankfurter Allgemeine Zeitung (FAZ), Süddeutsche Zeitung  Number of copies sold and coverage: FAZ 201,400 / 0.83 million readers; SDZ 311,000 / 1.3 million readers  Political orientation: FAZ (conservative), SDZ (left-liberal) | “Luftverschmutzung” (air pollution) or “Luftqualität” (air quality) in combination with “EU”  Total selected articles: 419  Random sample selected: 99  Number of statements: 585  Number of individuals: 202  Number of concepts: 29 |
| **Time frame**  We used the EU decision to sue Germany for non-compliance with its air pollution standards on 17^th^ May 2018 as the cutting point of the analysis. The time frame ranged from July 2015 to December 2020. | |
| **Additional sources**  **Archival research** of the following available documents pertaining to the identified organizations in Stuttgart and Berlin: air-pollution plans, reports on the development of air-pollution programs, and websites with information regarding air-pollution control or health risks posed by environmental pollution.  **Expert workshop**  A workshop with 31 experts in the field of air pollution in cities was held virtually in December 2020. These experts came from different disciplines, such as science institutes, city administrations, ministries, civil society organizations, NGOs, political parties, citizen science projects, health and medical organizations, as well as from the car industry and lobbying organizations.  **In-depth expert interviews**  We also conducted in-depth interviews with experts who served in organizations involved in the debates in Berlin (eight) and Stuttgart (nine) between February and December 2021 in order to verify the quality of our data. Interviewees were selected if they were frequently mentioned in the coded statements or if they were recommended by an expert as particularly relevant. These experts were drawn from different spheres of society, such as business, administration, politics, civil society, NGOs, and science. In these interviews, we enquired about the relevance of different actors in the debate to confirm that our analysis had not overlooked any important organizations.  The abovementioned sources were not the focus of the analysis; they only helped to verify the data quality and understand the context surrounding the study. | |
| **Coding process and inter-coder-reliability test**  The coding process started with a predefined list of concepts. This list was adjusted in an iterative process. One person was in charge of coding and followed a codebook created by the authors of the paper. During several rounds of data-cleaning and re-coding, we merged and defined concepts as well as types of actors/organizations. This process helped us make sure that concepts were clearly defined and did not overlap.  To check the reliability of the data, a second coder randomly selected a number of statements and verified that the coding scheme and the categories were consistent. Inconsistencies were discussed and refinements were made accordingly. This process continued until no further inconsistencies remained. We followed the DNA coding procedure of Buckton et al. (2019). | |
| **Salience and frequency of issues and actors** in the debate were measured according to the number of times that each actor/issue appeared in the coded newspaper articles. This measure of salience is biased due to media coverage tendencies and other influencing factors, such as the preferences of journalists or media sales restrictions.  **Identification of storylines**  According to Hajer and Versteeg (2005: 177), actors use simple storylines as “short hand” in debates and assume that others will understand what they mean. The assumption of mutual understanding is often misleading, due to the high complexity of the debated issues (ibid.). Actors share a specific set of storylines that serve to strengthen their own coalition and to create a sense of common understanding. In our analysis, we coded the concepts and examined whether certain actors link these concepts. If this was the case, they could be part of one (or more) consistent storyline(s) of a discourse coalition. | |
| **Normalization**  We used average activity normalization, which is the most commonly applied form of normalization and which works with binary and weighted *X* arrays, i.e., with or without duplicate statements. This measure divides each weight by the mean of the number of second-variable referrals of nodes *i* and *i’* (Leifeld, Gruber and Bossner, 2019: 8). | |
| **Network measures**  **Actor centrality**: According to Wasserman and Faust (1994: 173), central nodes are prominent actors that are extensively involved in relationships with other actors. This makes them more visible to other network actors. It does not matter if the prominence of the actor stems from incoming or outgoing ties, the focus is rather placed on the involvement of the actor in the network.  **Degree centrality** (as explained in detail by Wasserman and Faust 1994: 178–183): this centrality measure for an individual actor is equivalent to the activity of the node. We define $C_{D}(n_{i})$ as an actor-level degree centrality index, with the formula:  $C_{D}\left( n_{i} \right)= d\left( n_{i} \right)=x_{i+}= \sum_{j} x_{ij}= \sum_{j} x_{ji}$  The measure depends on group size *g.* Its maximum value is *g – 1*. Therefore, the proposed standardized measure would be calculated by using the following formula:  ${C^{'}}_{D}\left( n_{i} \right)= \frac{d(n_{i})}{g-1}$ | |

**3) Categorization of Actors According to the Type of Organization**

Following the manual categorization process explained above, actors were assigned attributes based on their organizational affiliation and activity at a particular level of governance. These categories are defined as follows:

**Type of organization**

*Government/Administration*

These organizations belong to the government and administration of a city, region, state, or nation. Examples of these are: ministries (Bund or Länder), authorities, courts, and all organizations affiliated and led by governmental actors. Organizations affiliated to the EU-governmental system, such as the European Environmental Agency (EEA), are part of this category too.

*NGO*

Here we have included non-governmental actors with the legal status “e.V.” (eingetragener Verein/registered association), except for those with a clear scientific mission (which have been coded as “Science”). The German legal status “e.V.” indicates that these organizations are not profit-oriented. Hence, we have included non-governmental organizations which are rather socially or environmentally oriented (such as BUND for environmental issues) as well as lobby groups (e.g., ADAC). Trade unions representing employees are also covered in this category.

*Politics*

This category includes political parties and other political organizations (such as think tanks/organizations affiliated with/working for political parties). It also applies to the representatives of a political party within a governmental institution (e.g., Bundestag MPs affiliated to the Green Party).

*Science*

We categorize here scientific research institutes, universities, and other independent or state-funded research institutes or think tanks that are neither profit-oriented nor part of governmental or political organizations. Their main task is the production of scientific studies. This category also includes individuals or organizations in the health and medical sectors (e.g., the “Eidgenössische Kommission für Lufthygiene”, which is an extra-parliamentary commission/committee that supplies governments with scientific expertise). Some organizations with the legal status “e.V.” are also included here, provided that their primary task consisted of the production of scientific knowledge (e.g., Leopoldina).

*Grassroots initiatives*

This category covers social movements as well as citizen initiatives that bring together groups of individuals with similar interests. Their organization is not as formal as that of NGOs. Single, private individuals expressing their views in public debates are included here, too.

*Economy*

This category contains all economic actors, private firms, and business associations with a clear profit-maximizing focus. This category also contains environmentally oriented economic actors, such as “Green City Solutions”.

*Public-sector economy*

All organizations from the public sector with some degree of private participation, such as BVG (a public-funded transit company headquartered in Berlin), are included in this category. The logic in this area is not primarily profit maximization, but rather the provision of a public service for the population.

*International organization*

International organizations with a clear focus on transboundary action and networking, such as the WHO or other UN bodies. These (generally) intergovernmental bodies have been established by a treaty and possess their own legal personality.

**Level of governance**

In order to analyze the affiliation of actors in the multi-level governance system, we have additionally coded and categorized the data based on their activity at different levels of governance: local, regional, state, national, EU, and international.

The categorization of actors was in some cases difficult, because some organizations are active on different levels. In this case, we have tried to identify their most important level of action and have ignored the other levels (e.g., DUH was categorized as an actor that is primarily active at the national level). In the cases where an organization operated with equal intensity at various levels of governance, we assigned the statement in question to the superior level. These decisions were made in order to simplify the analysis, although this could lead to distortions. We must bear this limitation in mind when interpreting the results.

**Two-mode visualizations**

**Figures A1–A3.** These are two-mode networks of organizations (circles) and concepts (squares). Organizations and concepts are tied by lines with different colors. Thus, green lines show congruent opinions, red lines show conflicting opinions, and blue lines indicate balanced opinions — namely that the same actor has expressed both congruent and conflicting opinions regarding the issue in question. We deleted nodes with a lower degree centrality in order to facilitate the interpretation of the results. These figures show the same node color scheme as Figures 2 to 7.

**Figure A1: Two-mode network in Stuttgart**


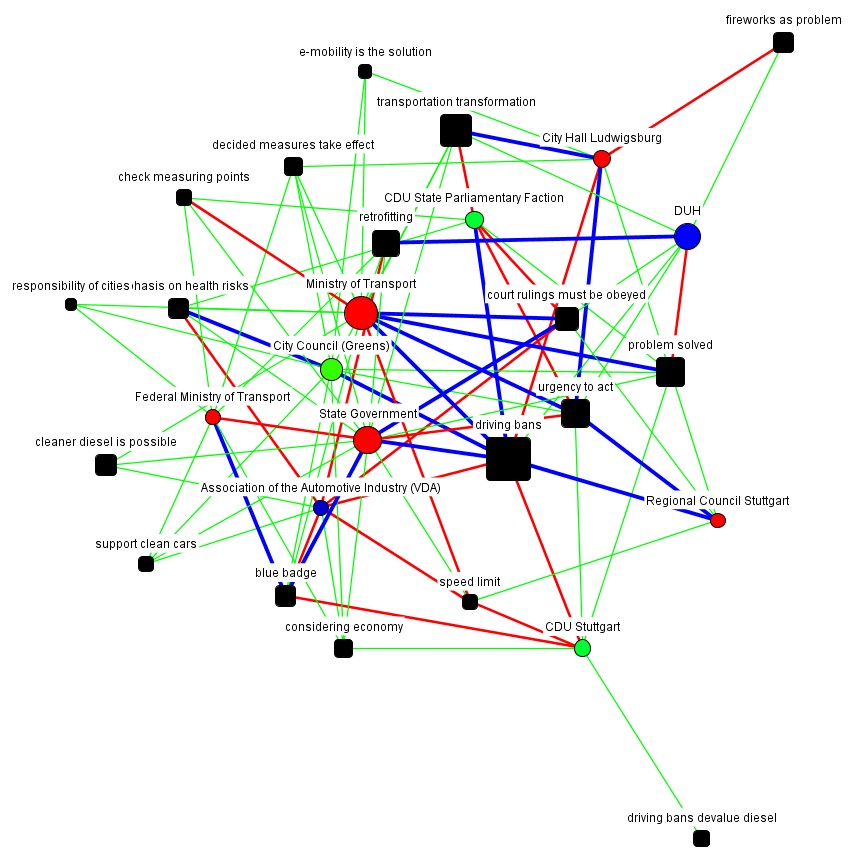


**Figure A2: Two-mode network in Berlin**


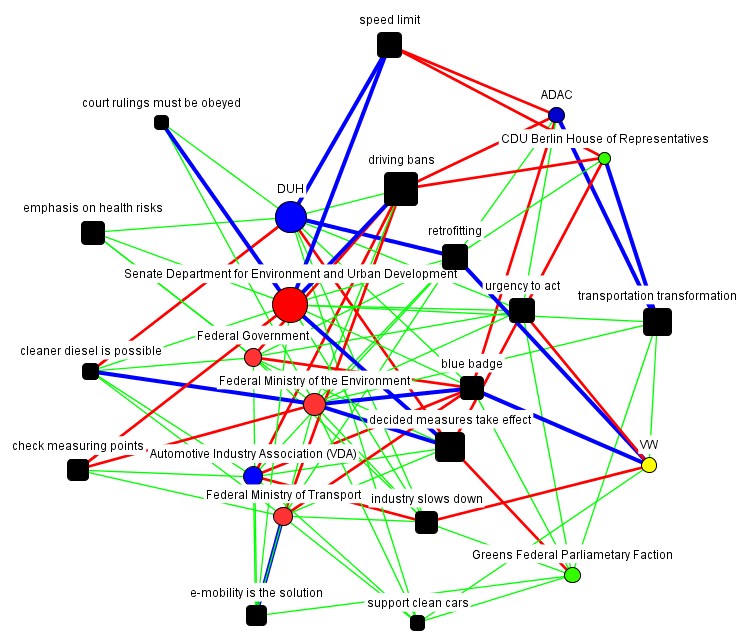


**Figure A3: Two-mode network in Brussels**


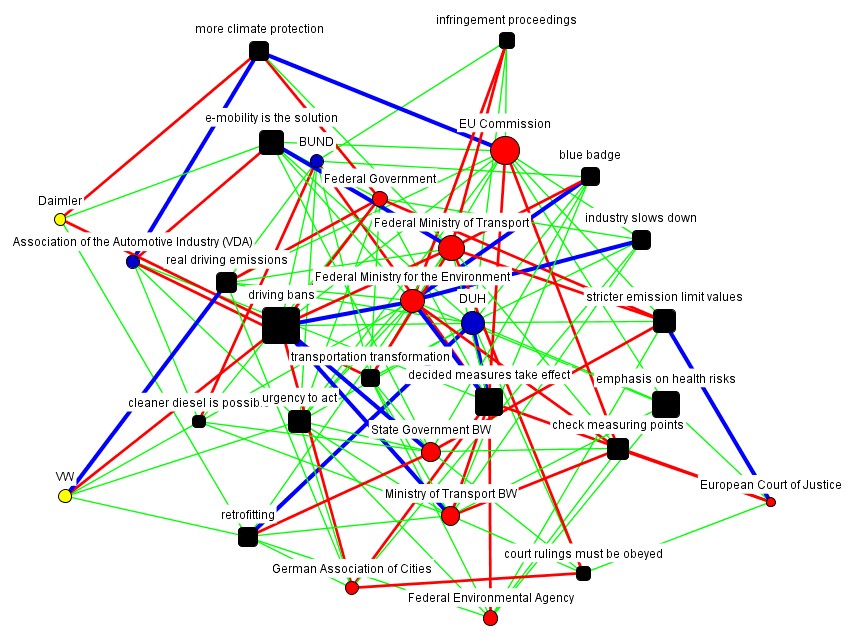


**References**

Buckton, C. H., Fergie, G., Leifeld, P., & Hilton, S. (2019). A Discourse Network Analysis of UK Newspaper Coverage of the “Sugar Tax” Debate before and after the Announcement of the Soft Drinks Industry Levy. *BMC Public Health* 19, 490

Hajer, M. and Versteeg, W. (2005) A Decade of Discourse Analysis of Environmental Politics: Achievements, Challenges, Perspectives, *Journal of Environmental Policy & Planning*, 7(3): 175–184, DOI: 10.1080/15239080500339646

Leifeld, P., Gruber, J. & Bossner, F. (2019). Discourse Network Analyzer Manual. Last Update: dna-2.0-beta25.jar with rDNA 2.1.18. September 11, 2019. Retrieved from: https://github.com/leifeld/dna /releases

Wasserman, S., & Faust, K. (1994). *Social Network Analysis: Methods and Applications*. Cambridge: Cambridge University Press

1. <https://www.tagesspiegel.de/themen/presseportal/leseranalyse-berlin-leseranalyse-berlin-2020-der-tagesspiegel-steigert-reichweite-um-17-prozent-und-ist-damit-die-reichweitenstaerkste-tageszeitung-in-der-hauptstadtregion/26961790.html> [↑](#footnote-ref-1)
2. <https://de.statista.com/statistik/daten/studie/1071746/umfrage/auflage-der-berliner-zeitung/> [↑](#footnote-ref-2)
3. The sample of the Stuttgart articles is twice as large as the remaining articles. We decided to increase this sample after realizing that the two selected newspapers occasionally published identical pieces. After removing all duplicate articles, we drew a random sample of articles from both newspapers. [↑](#footnote-ref-3)
